# Progressive Supranuclear Palsy: Improvement in Cognitive-Behavioral Disturbances and Motor-Function Disabilities Following Treatment With Antidepressants and Cholinesterase Inhibitors

**DOI:** 10.7759/cureus.15641

**Published:** 2021-06-14

**Authors:** Michelle Oyeka, Terngu Ibilah, Jacob Israel, Jose Gavito-Higuera, Ricardo Salazar

**Affiliations:** 1 Psychiatry, Texas Tech University Health Sciences Center El Paso Paul L. Foster School of Medicine, El Paso, USA; 2 Psychiatry/Geriatric Psychiatry, El Paso Veterans Affairs Health Care System, El Paso, USA; 3 Radiology/Neuroradiology, Texas Tech University Health Sciences Center El Paso Paul L. Foster School of Medicine, El Paso, USA; 4 Psychiatry/Geriatric Neuropsychiatry, Texas Tech University Health Sciences Center El Paso, EL Paso, USA; 5 Psychiatry/Geriatric Psychiatry, Brigham and Women's Hospital, Harvard Medical School, Boston, USA

**Keywords:** tauopathy, hispanics, progressive supranuclear palsy, antidepressants, cholinesterase inhibitors

## Abstract

Progressive supranuclear palsy (PSP) is a neurodegenerative disease that usually develops after the sixth decade of life, and the diagnosis is purely clinical except in cases of pathologically confirmed autopsies. A multidisciplinary approach to meet the patients’ complex needs is the current core treatment strategy for this devastating disorder. No medications can reverse the disease course. In this report, we present a case of PSP that developed after the sixth decade of life and where the diagnosis was supported by clinical and neuroimaging data. Despite the fact that PSP is a rapidly progressive neurodegenerative disorder and no effective treatments are currently available, our case illustrates the clinically significant improvement in cognition and function achieved in a patient with a treatment involving a combination of antidepressant medications and rivastigmine.

## Introduction

Progressive supranuclear palsy (PSP) is a neurodegenerative disease usually seen in individuals after the sixth decade of life, and the diagnosis is either clinical or post-mortem following a pathologically confirmed autopsy. The onset of PSP is insidious and usually includes a prolonged phase marked by the following symptoms: fatigue, headaches, arthralgias, dizziness, depression, subtle personality changes, memory problems, and pseudobulbar symptoms, which are often more evident to the family. The initial symptoms are sometimes unexplained imbalance or falls [1]. Steele, Richardson, and Olszewski designated PSP as a new clinicopathological entity in their seminal paper published more than 60 years ago [2]. Since then, in addition to the classic Richardson’s syndrome (RS), various clinical phenotypic presentations have been linked with this four-repeat tauopathy [aggregates of four-repeat tau in astrocytes (tufts), oligodendrocytes (coiled bodies), and neurons (neurofibrillary tangles) in typical distribution that predominates in the basal ganglia and brainstem]. The clinical heterogeneity is associated with variability of regional distribution and severity of abnormal tau accumulation and neuronal loss [3]. In PSP subtypes, the presence of certain clinical pointers may be useful for antemortem prediction of the underlying PSP-tau pathology. Midbrain atrophy on conventional MRI correlates with the clinical phenotype of RS but is not predictive of PSP pathology. Cerebrospinal fluid biomarkers and tau ligand positron emission tomography are promising biomarkers of PSP [4,5]. A multidisciplinary approach to meet the patients’ complex needs is the current core treatment strategy for this devastating disorder. No medications can reverse the disease course. We discuss the case of a patient who developed the condition after the sixth decade of life; clinical and neuroimaging data confirmed the diagnosis. Despite PSP being a rapidly progressive neurodegenerative disorder and no effective treatments are currently available, our case highlights the clinically significant improvement in cognition and function that was achieved with a treatment modality characterized by a combination of antidepressant medications and rivastigmine.

## Case presentation

The patient was a Hispanic male in his 70s with no previous history of psychiatric diseases who had been referred to our memory clinic from neurology. He had initially presented to a neurology clinic in August of 2018 with a self-reported history of gait instability with frequent falls, hypophonia, and trouble remembering recent events, which had been documented on his neurological exam. The patient stated that he had developed progressive and persistent issues with swallowing as well as fecal and urinary incontinence two years prior to the current presentation, The differential diagnosis at the time of his first neurology visit had been highly concerning for olivopontocerebellar atrophy (OPCA), along with other forms of atypical parkinsonism including dementia with Lewy bodies (DLB), multiple system atrophy (MSA), and corticobasal degeneration (CBD), which were closely associated with OPCA, particularly MSA. A diagnosis of DLB had been ruled out due to the absence of visual hallucinations and cognitive deterioration as a presenting symptom and, later on, parkinsonism. In MSA, the main clinical feature includes autonomic dysfunction combined with cerebellar ataxia (which was absent in our patient) or parkinsonism. Finally, CBD as a more uncommon syndrome typically manifests itself as markedly asymmetrical parkinsonism (not present in our patient) with apraxia (present in our patient) or cortical sensory disturbances (not present in our patient). 

At a follow-up two months later, the patient's symptoms had considerably worsened, and he had shown notable difficulty in getting up from chairs due to muscle rigidity and bradykinesia, as well as dragging of his feet. Laboratory investigations including vitamin B12, folate, methylmalonic acid, homocysteine, vitamin E, vitamin D, copper levels, HIV antibody, and human T-cell leukemia viruses 1 (HTLV-1)/HTLV-2 had all been within normal limits.

**Figure 1:**
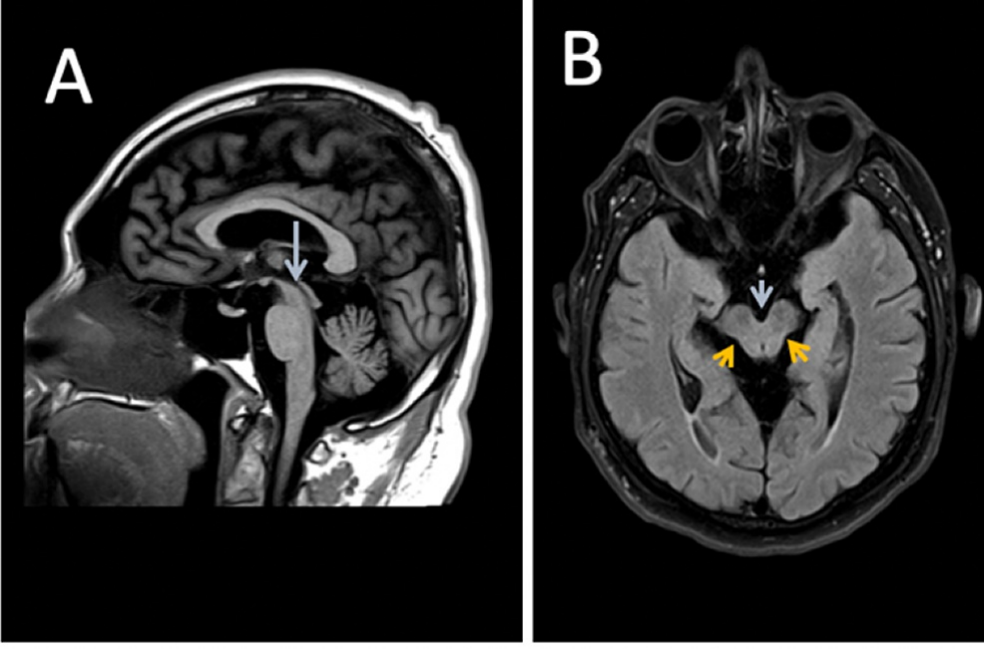
Neuroimaging findings in the patient Panel A: sagittal T1-weighted sequence shows prominent atrophy of the midbrain and superior colliculus, resulting in the concavity of the cranial mesencephalic margin. There is a "hummingbird sign" due to preserved pontine volume. Findings are most consistent with PSP. Panel B: axial fluid-attenuated inversion recovery (FLAIR) sequence MRI also demonstrates a concave dorsolateral midbrain margin. The corresponding axial images reveal a concave dorsolateral midbrain margin (orange arrows) and widening of the interpeduncular cistern (blue arrow) MRI: magnetic resonance imaging; PSP: progressive supranuclear palsy

A second MRI in November of 2018 had shown prominent atrophy of the midbrain with mild diffuse cerebellar and cerebral volume loss, and "superior colliculus with the convexity of the cranial mesencephalic margin on sagittal view", also known as the "hummingbird sign" (Figure 1). 

At that time, the patient had been diagnosed with PSP, which had correlated with his clinical presentation and neurological exam that included documentation of slowed oculomotor function with hypometric vertical saccades (please see summary, Table 1). Subsequently, the patient had been referred to a geriatric neuropsychiatry clinic for an expert consultation with a geriatric psychiatrist for the further management of his neuropsychiatric, motor, and cognitive symptoms.

**Table 1:** Summary of various factors related to PSP PSP: progressive supranuclear palsy; MRI: magnetic resonance imaging; PPV: positive predictive value Adapted from Levin et al., 2016 [5]

Progressive supranuclear palsy	
Areas	Findings/recommendations
Neuropathology	Aggregates of four-repeat tau in astrocytes (tufts), oligodendrocytes (coiled bodies), and neurons (neurofibrillary tangles) in typical distribution (predominant in basal ganglia and brainstem)
Clinical syndrome	Richardson's syndrome (PSP-RS, about 40% of cases, PPV: about 90%): symmetric, axial-oriented, akinetic-rigid, levodopa-resistant parkinsonian syndrome with early postural instability and vertical supranuclear gaze palsy. PSP with predominant parkinsonian symptoms (PSP-P, about 20% of cases, PPV: about 90%): asymmetric, limb-predominant, levodopa-responsive parkinsonian syndrome with late-onset vertical supranuclear gaze palsy. Behavioral variant of frontotemporal dementia (bvFTD, about 15% of cases, PPV: low): apathy and impaired executive functions (<6 successive Luria sequences, applause sign), late-onset vertical supranuclear gaze palsy. Corticobasal syndrome (CBS, about 10% of cases, PPV: about 30%): at least one cortical symptom (apraxia, loss of cortical sensitivity, alien limb phenomenon) and at least one extrapyramidal symptom (akinesia, rigidity, dystonia, myoclonus). Progressive non-fluent aphasia (PNFA, about 5% of cases, PPV: low): non-fluent speech production (<9 words beginning with S in 60 seconds) with agrammatism but spared single-word comprehension. Pure akinesia with gait freezing (PAGF, <5% of cases, PPV: about 60%): gait-freezing without rigidity, without tremors, late-onset vertical supranuclear gaze palsy
MRI results	Midbrain atrophy: axial anteroposterior diameter <15 mm; frontal lobe atrophy. Exclude symptomatic causes: vascular encephalopathy, normal pressure hydrocephalus, aqueductal stenosis, encephalitis, tumor
Neurological exam	Oculomotor function: slowed, hypometric vertical saccades, frequent square wave jerks
Levodopa test	Poor responsiveness to levodopa
Symptomatic treatment	Poor evidence

During the patient’s initial psychiatric presentation, he had been noted to have a diminished sense of purpose, fatigue, excessive sleepiness, lack of motivation as well as feelings of "crumbling manhood" and a sense of helplessness with insidious onset in the past six months. At that time, he had also disclosed increased difficulty in remembering the names of family and friends and increased difficulty in finding appropriate words. The following symptoms of parkinsonism had also been observed at that time: masked facies, bradykinesia, and shuffling gait.

**Figure 2:**
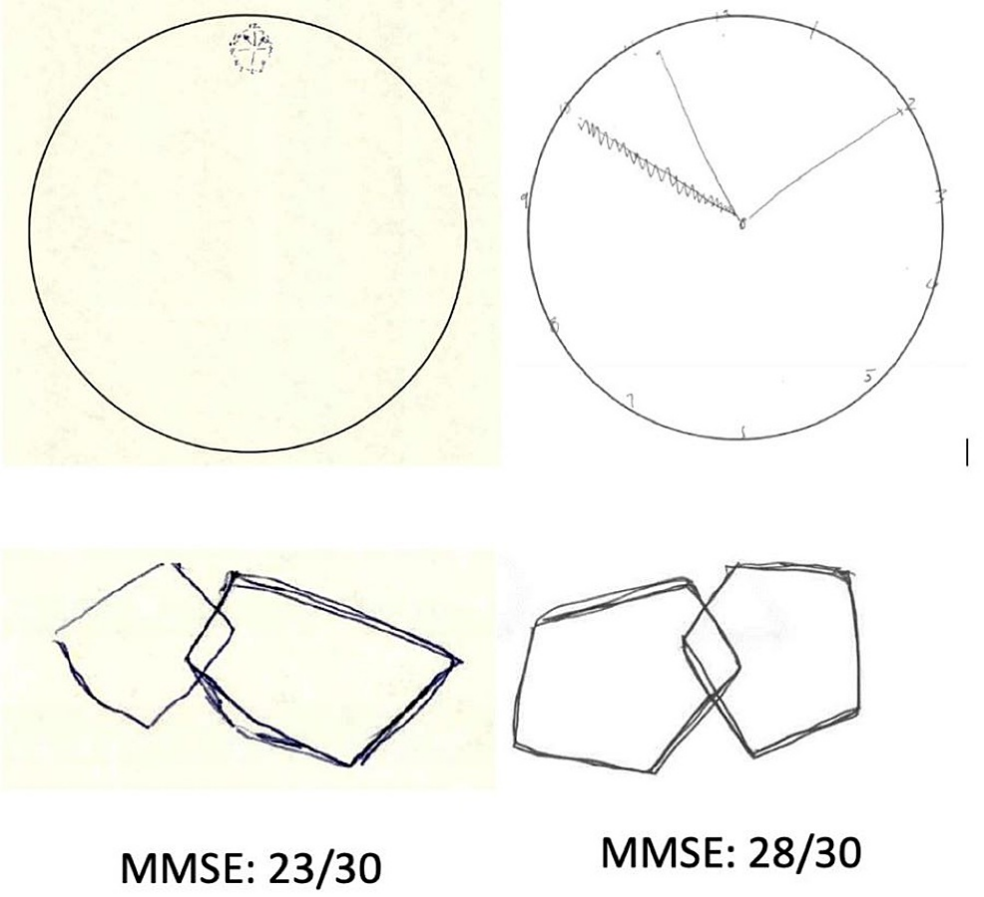
Cognitive performance findings in the patient Baseline and five-month post-treatment cognitive performance in a test of global cognition, Mini-Mental State Examination (MMSE) and the Alzheimer's Association clock-drawing task. At baseline, the patient showed prominent constructional apraxia and dysexecutive impairment with micrographic clock-drawing performance and poor pentagon drawing. After starting treatment with rivastigmine 1.5 mg PO BID, escitalopram 10 mg PO daily, and bupropion XL 150 mg PO daily, motor, praxis, and dysexecutive impairment with five-point increment on MMSE was achieved and correlated with behavioral and mood symptom improvement

The patient was administered a Mini-Mental State Examination (MMSE), and he demonstrated pronounced micrographia and other abnormalities (Figure 2). He was diagnosed with PSP-parkinsonism (PSP-P) and major depressive disorder. He was started on rivastigmine 1.5 mg and escitalopram 10 mg every day in the morning (selective serotonin reuptake inhibitor), and bupropion XL 150 mg daily (norepinephrine dopamine reuptake inhibitor). In this way, we targeted cognitive/motor disabilities and mood symptoms respectively, which justified the use of two synergistic antidepressants to influence and stimulate the serotonin, norepinephrine, and dopamine pathways.

With continued treatment, speech therapy, and physical therapy, the patient’s symptoms continued to improve. His escitalopram was discontinued as he reported that he was not regularly taking it due to the side effects of excessive fatigue and sleepiness. Most of his initial symptoms have now stabilized, and there has been considerable improvement in his fine motor movements as demonstrated on his clock-drawing test. However, he continues to struggle with his shuffling and retropulsion gait, characterized by frequent backward falls. Nevertheless, the patient’s mood has greatly improved.

## Discussion

PSP is a rapidly progressive neurodegenerative disease classically identified by its predominant feature of supranuclear gaze palsy and nerve cell degeneration mainly noted in the brain stem [1]. However, a definitive diagnosis of PSP requires post-mortem neuropathological examination, particularly to identify intracerebral aggregation of the microtubule-associated protein tau spread out in neurofibrillary tangles, oligodendrocytic coils, and astrocyte tufts [1]. These abnormally phosphorylated four-repeat tau proteins can be found distributed throughout the globus pallidus, subthalamic nucleus, dentate nucleus, substantia nigra, midbrain, pons, and medulla oblongata [6].

Multiple sources have estimated the prevalence of PSP to be 5.8 to 6.5 cases per 100,000 people [3]. While there has been some speculation about the various factors that can contribute to PSP, the only reliably confirmed risk factor appears to be educational attainment, which may serve as a marker for "synaptic reserve" [7]. This observation was noted with our patient who had only attained a 10th-grade education. The exact cause of PSP remains unknown, with no discernible pattern of increased risk factors linked to race, ethnicity, or geographical location [7]. 

While the classical PSP, previously recognized as RS, is well-characterized, it only accounts for a small percentage of actual diagnosed cases. For example, Respondek et al. examined 100 autopsy-confirmed cases of PSP and noted that only 24% of those cases had actually presented as RS, while more than half of the cases had presented with overlapping features of various prescribed phenotypes that do not fit the classical description of PSP [6]. The main variants consistently described in the literature include PSP-P, PSP-pure akinesia with gait freezing (PSP-PAGF), PSP-progressive non-fluent aphasia/apraxia of speech (PSP-PNFA/AOS), PSP-cerebellar (PSP-C), PSP-behavioral variant frontotemporal dementia (PSP-bvFTD), and PSP corticobasal syndrome (PSP-CBS) [8].

Our patient presented with Parkinson's symptoms in addition to his previously established PSP, likely indicating a diagnosis of PSP-P. In this variant, patients often present clinically with less pronounced dementia, asymmetrical onset of tremors, early bradykinesia, and non-axial dystonia, which has been documented to be responsive to levodopa therapy [9]. According to Willams et al., bradykinesia was determined to be the most essential feature for a diagnosis of PSP-P. They also noted that the average disease duration of this variant was longer than that of classical PSP-RS, 9.1 years compared to 5.9 years, with the clinical features between PSP-P and PSP-RS generally more distinguishable within the first two years of diagnosis [9]. Our patient was found to be relatively stable with no notable worsening with regard to the decline of mood or dementia-like symptoms. As mentioned above, PSP-P and PSP-PAGF are associated with a more benign course of the disease process, longer survival periods, and a less tau protein burden overall compared to PSP-RS, with the abnormal tau protein relatively restricted to the brainstem [3]. This provides further support that our patient likely has a PSP-variant, which remains subcortical given his mild progression thus far. Furthermore, our patient presented with fewer symptoms that do not correlate with a cortical dementia presentation. He did not show any symptoms of rapid personality changes or non-fluent aphasia associated with PSP-bvFTD, PSP-PNFA, and PSP-CBS. In fact, his MMSE was relatively preserved, with his initial test scoring 30/30 at his first neurological visit, prior to his visit to the geriatric psychiatry clinic. Most of his symptoms appeared to present strongly with motor difficulties and mood dysregulation as opposed to severe cognitive impairment. It is important to note that the patient’s cognitive deficits seemed to abate with his improved mood.

After two years of following up on his original diagnosis, our patient is currently stable on a medication regimen that consists of two primary prescriptions, bupropion 150 mg XL and rivastigmine 1.5 mg. Currently, there is no established therapy for PSP. The use of dopaminergic agents discussed in the literature has been shown to provide limited efficacy [3,8].

Other treatments that have been previously utilized to address symptoms of rigidity and associated parkinsonian phenotypes include antiparkinsonian medications such as levodopa, and methysergide, which has been effective for ophthalmoplegia, pseudobulbar symptoms, and mental status [8,10,11].

It is interesting to note that while our patient was never started on levodopa to address the parkinsonism symptoms associated with the PSP, the low-dose bupropion has been instrumental in improving both his mood-related symptoms and associated parkinsonism; rivastigmine, which is an FDA-approved treatment for Parkinson's disease dementia, has also had a favorable impact. More studies are needed to explore the various combinations of disease-modifying drugs that can address the deterioration associated with PSP and its various phenotypical manifestations.

## Conclusions

PSP is a progressive neurodegenerative disease, and no treatments/medications to reverse the disease course are currently available. In our patient, the use of combined antidepressants in conjunction with cholinesterase inhibitors improved cognitive function and ameliorated motor and neuropsychiatric symptoms, thereby improving his quality of life.
